# Supplementary material for: The influence of fetal sex on maternal blood pressure in pregnancy
Source: BMC Med. 2025 Nov 5;23:612. doi: 10.1186/s12916-025-04432-0 (PMC12590762; doi:10.1186/s12916-025-04432-0)
Supplement: Supplementary file 1 — Additional file 1: Supplementary Tables S1 and S2. Table S1 The SNPs used to create the genetic scores. Table S2 The strength of association between the fetal genetic score for birth weight and measured offspring birth weight in each cohort. [file 12916_2025_4432_MOESM1_ESM.docx]

***Supplementary Information***

Supplementary Tables

**Supplementary Table S1. 186 autosomal own birth weight-associated lead SNPs, oriented to the increaser allele.** From the marginal analysis of Warrington et al. 2019 genome-wide association study for birth weight.

| **SNP** | **Chrom:position (hg19)** | **Trait-raising Allele** | **Trait-lowering Allele** | **Beta (SEM^a^ adjusted fetal effects)** |
| --- | --- | --- | --- | --- |
| rs17367504 | chr1:11862778 | A | G | 0.005 |
| rs12401656 | chr1:43456767 | G | A | 0.029 |
| rs80278614 | chr1:119412317 | A | G | 0.052 |
| rs905938 | chr1:154991389 | C | T | 0.023 |
| rs670523 | chr1:155878732 | G | A | 0.016 |
| rs72480273 | chr1:161644871 | C | A | 0.022 |
| rs10913200 | chr1:176521655 | G | A | 0.038 |
| rs61830764 | chr1:212289976 | A | G | 0.018 |
| rs3806315 | chr1:214724668 | A | G | 0.016 |
| rs708122 | chr1:228216997 | C | A | 0.015 |
| rs10495563 | chr2:9662210 | A | G | 0.016 |
| rs2551347 | chr2:23912401 | T | C | 0.029 |
| rs1179494 | chr2:36809496 | G | C | 0.002 |
| rs754868 | chr2:43185532 | G | A | 0.019 |
| rs4952673 | chr2:43423870 | G | A | 0.004 |
| rs17034876 | chr2:46484310 | T | C | 0.039 |
| rs4953353 | chr2:46567276 | G | T | 0.019 |
| rs186606513 | chr2:97482001 | G | A | 0.047 |
| rs56188432 | chr2:158406865 | G | A | 0.25 |
| rs560887 | chr2:169763148 | T | C | 0.025 |
| rs2280235 | chr2:191843830 | G | A | 0.014 |
| rs10181515 | chr2:227019461 | T | C | 0.021 |
| rs9855896 | chr3:14287150 | A | G | 0.014 |
| rs2168443 | chr3:46947087 | T | A | 0.01 |
| rs11708067 | chr3:123065778 | G | A | 0.056 |
| rs9851257 | chr3:123125711 | T | A | 0.005 |
| rs6440006 | chr3:141142691 | G | A | 0.001 |
| rs2306700 | chr3:142123841 | T | C | 0.022 |
| rs10935733 | chr3:148622968 | T | C | 0.021 |
| rs4679760 | chr3:155855418 | C | G | 0.009 |
| rs1482852 | chr3:156798294 | A | G | 0.054 |
| rs11711420 | chr3:183349010 | T | G | 0.022 |
| rs4144829 | chr4:17903654 | C | T | 0.032 |
| rs6533183 | chr4:106133184 | C | T | 0.008 |
| rs116807401 | chr4:135121721 | C | T | 0.088 |
| rs6845999 | chr4:145565826 | T | C | 0.017 |
| rs4579095 | chr4:174726635 | A | G | 0.007 |
| rs1818782 | chr5:39424628 | C | A | 0.015 |
| rs351930 | chr5:52003397 | T | A | 0.02 |
| rs854037 | chr5:57091783 | A | G | 0.02 |
| rs28365970 | chr5:67585723 | C | A | 0.015 |
| rs1981627 | chr5:133838180 | G | A | 0.007 |
| rs2946179 | chr5:157886627 | T | C | 0.004 |
| rs34471628 | chr5:172196752 | G | A | 0.014 |
| rs9379084 | chr6:7231843 | G | A | 0.004 |
| rs35261542 | chr6:20675792 | C | A | 0.049 |
| rs9379832 | chr6:26186200 | A | G | 0.019 |
| rs9366778 | chr6:31269173 | G | A | 0.014 |
| rs6911024 | chr6:31368451 | C | T | 0.002 |
| rs9267812 | chr6:32128394 | T | C | 0.015 |
| rs1547669 | chr6:33775641 | G | A | 0.018 |
| rs75104038 | chr6:34190104 | A | G | 0.024 |
| rs9348981 | chr6:35687249 | T | G | 0.015 |
| rs7744700 | chr6:53349401 | T | A | 0.018 |
| rs76094073 | chr6:109288036 | G | C | 0.011 |
| rs6925689 | chr6:126865884 | T | C | 0.018 |
| rs6569647 | chr6:130337266 | T | C | 0.014 |
| rs6930558 | chr6:141878920 | T | G | 0.022 |
| rs962554 | chr6:142734204 | T | C | 0.015 |
| rs10872678 | chr6:152039964 | T | C | 0.028 |
| rs2934844 | chr6:166142456 | T | A | 0.018 |
| rs4719648 | chr7:2756832 | C | T | 0.014 |
| rs59084784 | chr7:22739562 | A | C | 0.011 |
| rs7808457 | chr7:22798265 | A | T | 0.002 |
| rs34776209 | chr7:23513093 | C | T | 0.015 |
| rs2908279 | chr7:44174857 | T | G | 0.007 |
| rs2971669 | chr7:44231778 | C | T | 0.003 |
| rs138715366 | chr7:44246271 | C | T | 0.235 |
| rs10265133 | chr7:45895604 | T | G | 0.02 |
| rs11983722 | chr7:46298647 | A | T | 0.029 |
| rs10265057 | chr7:47275737 | G | A | 0.036 |
| rs2237467 | chr7:50733316 | A | G | 0.011 |
| rs112139215 | chr7:73034559 | A | C | 0.056 |
| rs2282978 | chr7:92264410 | C | T | 0.021 |
| rs45446698 | chr7:99332948 | T | G | 0.017 |
| rs6467157 | chr7:127660763 | T | C | 0.014 |
| rs3918226 | chr7:150690176 | T | C | 0.005 |
| rs62496903 | chr8:6446938 | T | C | 0.028 |
| rs732563 | chr8:23345526 | C | T | 0.019 |
| rs11778247 | chr8:23403378 | A | G | 0 |
| rs34036147 | chr8:38366249 | T | C | 0.019 |
| rs13266210 | chr8:41533514 | A | G | 0.03 |
| rs72656010 | chr8:57122215 | T | C | 0.026 |
| rs6995390 | chr8:77611012 | A | T | 0.014 |
| rs7819593 | chr8:106115172 | C | T | 0.023 |
| rs10283100 | chr8:120596023 | G | A | 0.033 |
| rs13271368 | chr8:126506140 | C | T | 0.021 |
| rs13257363 | chr8:142252580 | G | A | 0.017 |
| rs9657468 | chr8:142362391 | G | T | 0.018 |
| rs7854962 | chr9:96900505 | C | G | 0.016 |
| rs28457693 | chr9:98217348 | G | A | 0.04 |
| rs2418135 | chr9:113901309 | A | G | 0.012 |
| rs72760655 | chr9:116916214 | A | C | 0.009 |
| rs1323438 | chr9:119115531 | C | T | 0.02 |
| rs3933326 | chr9:123633948 | G | A | 0.023 |
| rs10985827 | chr9:125701608 | G | T | 0.027 |
| rs28505901 | chr9:139241030 | A | G | 0.024 |
| rs4350272 | chr10:25056118 | A | G | 0.017 |
| rs9645500 | chr10:70986723 | G | T | 0.019 |
| rs1112718 | chr10:94479107 | G | A | 0.036 |
| rs10509669 | chr10:95969913 | T | A | 0.02 |
| rs3740360 | chr10:96025491 | C | A | 0.003 |
| rs2274224 | chr10:96039597 | C | G | 0.019 |
| rs562974282 | chr10:104201070 | T | G | 0.126 |
| rs10883846 | chr10:104958244 | C | T | 0.016 |
| rs7903146 | chr10:114758349 | T | C | 0.003 |
| rs7076938 | chr10:115789375 | T | C | 0.029 |
| rs71486610 | chr10:124134803 | C | G | 0.016 |
| rs11042596 | chr11:2118860 | T | G | 0.027 |
| rs234864 | chr11:2857297 | A | G | 0.017 |
| rs2168101 | chr11:8255408 | A | C | 0.015 |
| rs4444073 | chr11:10331664 | A | C | 0.023 |
| rs5030317 | chr11:32410337 | C | G | 0.007 |
| rs10437653 | chr11:46297631 | A | C | 0.002 |
| rs10734564 | chr11:48160429 | G | A | 0.009 |
| rs667515 | chr11:69449076 | G | C | 0.013 |
| rs61885091 | chr11:69791952 | A | G | 0.024 |
| rs10830963 | chr11:92708710 | C | G | 0.002 |
| rs10895278 | chr11:102095335 | T | C | 0.001 |
| rs76895963 | chr12:4384844 | G | T | 0.051 |
| rs11055030 | chr12:12878349 | G | C | 0.022 |
| rs2306547 | chr12:26877885 | C | T | 0.016 |
| rs11051061 | chr12:30914668 | A | G | 0.001 |
| rs6582623 | chr12:46613394 | C | T | 0.02 |
| rs180438 | chr12:47187260 | A | G | 0.007 |
| rs7968682 | chr12:66371880 | G | T | 0.037 |
| rs1480470 | chr12:66412130 | G | A | 0.028 |
| rs1533688 | chr12:102772745 | T | C | 0.004 |
| rs2647873 | chr12:103081192 | A | G | 0.009 |
| rs17033114 | chr12:103123339 | C | T | 0.008 |
| rs3184504 | chr12:111884608 | C | T | 0.005 |
| rs9549046 | chr13:40647206 | A | G | 0.027 |
| rs34217484 | chr13:48854550 | A | T | 0.012 |
| rs9318511 | chr13:78601413 | C | A | 0.024 |
| rs72681869 | chr14:50655357 | C | G | 0.108 |
| rs6575803 | chr14:101257755 | C | T | 0.034 |
| rs75844534 | chr15:38667117 | A | C | 0.036 |
| rs2928148 | chr15:41401550 | G | A | 0.004 |
| rs339969 | chr15:60883281 | A | C | 0.011 |
| rs3784789 | chr15:75082552 | C | G | 0.018 |
| rs12909648 | chr15:86224570 | A | G | 0.003 |
| rs12443252 | chr15:91064690 | C | T | 0.007 |
| rs4932373 | chr15:91429287 | A | C | 0.01 |
| rs55958435 | chr15:96852638 | A | G | 0.022 |
| rs7402983 | chr15:99193276 | A | C | 0.027 |
| rs11630479 | chr15:99240481 | G | A | 0.007 |
| rs2045457 | chr16:20046115 | G | A | 0.012 |
| rs40434 | chr16:55699525 | G | A | 0.017 |
| rs28544888 | chr16:55741204 | C | T | 0.027 |
| rs11641308 | chr16:75312023 | C | T | 0.005 |
| rs222857 | chr17:7164563 | T | C | 0.026 |
| rs4511593 | chr17:7455536 | T | C | 0.019 |
| rs78378222 | chr17:7571752 | G | T | 0.058 |
| rs9909342 | chr17:25652275 | A | G | 0.019 |
| rs7223535 | chr17:29211667 | G | A | 0.02 |
| rs11867479 | chr17:68090207 | T | C | 0.018 |
| rs10221267 | chr17:68464662 | T | C | 0.018 |
| rs73354194 | chr17:79905947 | C | T | 0.06 |
| rs9912553 | chr17:79959703 | G | C | 0.006 |
| rs11082304 | chr18:20720973 | T | G | 0.013 |
| rs2779165 | chr19:4915447 | G | C | 0.018 |
| rs8106042 | chr19:7161849 | G | C | 0.023 |
| rs2967676 | chr19:8789666 | C | A | 0.003 |
| rs41355649 | chr19:33790556 | G | A | 0.042 |
| rs1129156 | chr19:40719076 | T | C | 0.022 |
| rs147957154 | chr19:43431040 | T | C | 0.026 |
| rs516246 | chr19:49206172 | C | T | 0.017 |
| rs255773 | chr19:54723546 | C | T | 0.018 |
| rs147110934 | chr19:55993436 | G | T | 0.055 |
| rs12461110 | chr19:56320663 | G | A | 0.005 |
| rs304001 | chr19:56423668 | A | G | 0.003 |
| rs6040076 | chr20:10658882 | C | G | 0.015 |
| rs6033062 | chr20:11207419 | A | T | 0.014 |
| rs1203876 | chr20:22540915 | C | A | 0.055 |
| rs11698914 | chr20:31327144 | C | G | 0.029 |
| rs181451002 | chr20:32466219 | A | G | 0.006 |
| rs2889874 | chr20:33715777 | G | T | 0.014 |
| rs1012167 | chr20:39159119 | C | T | 0.024 |
| rs753381 | chr20:39797465 | T | C | 0.018 |
| rs6026449 | chr20:57272617 | C | T | 0.018 |
| rs73143584 | chr20:62445702 | A | G | 0.031 |
| rs2229742 | chr21:16339172 | G | C | 0.028 |
| rs220193 | chr21:43581308 | A | G | 0.018 |
| rs134594 | chr22:29468456 | C | T | 0.022 |
| rs41311445 | chr22:42070374 | A | C | 0.034 |
| rs7285579 | chr22:46441980 | C | T | 0.018 |

^a^SEM = structural equation model for partitioning of maternal and fetal effects. Note: genetic scores were unweighted i.e. all SNP weights set to 1 for analysis

**Supplementary Table S2. The strength of association between the fetal genetic score for birth weight and measured offspring birth weight in each cohort.** ALSPAC, Avon Longitudinal Study of Parents and Children; BiB, Born in Bradford study; EFSOCH, Exeter Family Study of Childhood Health; HAPO, Hyperglycemia and Adverse Pregnancy Outcome study; MoBa, Norwegian Mother, Father and Child Cohort Study.

| **Cohort** | **n** | **Effect/Coefficient*** | **SE** | **95% CI** | **P-value** | **R^2^** |
| --- | --- | --- | --- | --- | --- | --- |
| MoBa† | 23,065 | 85.9 | 3.06 | 79.9 - 91.9 | 2.87x10^-170^ | -- |
| EFSOCH | 539 | 63.6 | 20.20 | 23.9 – 103.3 | 0.0017 | 0.0181 |
| HAPO | 785 | 51.9 | 14.06 | 24.3 - 79.5 | 2.4x10^-4^ | 0.0171 |
| ALSPAC | 5,194 | 80.3 | 6.40 | 67.8 – 92.9 | 1.3x10^-35^ | 0.0294 |
| BiB - European | 1,804 | 61.2 | 12.53 | 36.7 – 85.8 | 1.1x10^-6^ | 0.0131 |
| BiB - Pakistani | 1,605 | 56.6 | 11.88 | 33.2 – 79.9 | 2.1x10^-6^ | 0.0139 |
| BiB - Bangladeshi | 66 | -18.8 | 56.08 | -130.9 – 93.2 | 0.74 | 0.0018 |

*The effect represents the change in offspring birth weight (g) per 1 standard deviation change in fetal birth weight genetic score

†Association between a weighted genetic score with offspring birth weight
