# Supplementary material for: The influence of fetal sex on maternal blood pressure in pregnancy
Source: BMC Med. 2025 Nov 5;23:612. doi: 10.1186/s12916-025-04432-0 (PMC12590762; doi:10.1186/s12916-025-04432-0)
Supplement: Supplementary file 2 — Additional file 2: Supplementary Note. Power calculations. [file 12916_2025_4432_MOESM2_ESM.docx]

Supplementary Note

**Power Calculations**

Calculations performed in R to estimate the power to detect the odds ratio observed in our analysis of the effect of fetal sex on maternal high blood pressure (OR 1.05 [95%CI: 0.98-1.12]), and to estimate the power to identify effect sizes equivalent to an established binary risk factor for high blood pressure, such as overweight or obesity. We used a formula for noncentrality parameters adapted from Appendix A of Vukcevic et al. (Genet. Epidemiol. 2011, 35(4):278-290; <https://doi.org/10.1002%2Fgepi.20576>).

####### Power Calculations #######

#Formula for NCP (noncentrality parameters) adapted from Appendix A of Vukcevic et al 2011

#i.e. NCP <- N*phi*(1-phi)*(log(OR))^2*p*(1-p)

#where N is total number of individuals

#phi is proportion of cases

#OR is odds ratio

#p is the probability of being male

#Have substituted p*(1-p) into the Vukcevic formula as this the variance of a bernoulli sex variable (i.e. instead of variance of SNP under HWE)

# Estimating Power with the OR found in our study

ncases <- 3820

ncontrols <- 106022

phi <- ncases/(ncases+ncontrols)

N <- 109842

p <- 0.5 #Frequency of male sex

OR <- 1.05

NCP <- N*phi*(1-phi)*(log(OR))^2*p*(1-p) #Vukcevic formula adapted

pchisq(q=3.84, df=1, ncp = NCP, lower.tail = FALSE, log.p = FALSE) #Power of ~32%

# Estimating Power with OR of comparable meaningful effect size

# e.g. 1.7 higher odds of having hypertensive disorders in pregnancy for women who were overweight compared to normal weight according to their pre-pregnancy BMI

# from Nohr et al. (Am J Clin Nutr. 2008, 87(6):1750-1759; https://doi.org/10.1093/ajcn/87.6.1750)

ncases <- 3820

ncontrols <- 106022

phi <- ncases/(ncases+ncontrols)

N <- 109842

p <- 0.5 #Frequency of male sex

OR <- 1.7

NCP <- N*phi*(1-phi)*(log(OR))^2*p*(1-p) #Vukcevic formula adapted

pchisq(q=3.84, df=1, ncp = NCP, lower.tail = FALSE, log.p = FALSE) #Power of 100%
