# Supplementary material for: The influence of fetal sex on maternal blood pressure in pregnancy
Source: BMC Med. 2025 Nov 5;23:612. doi: 10.1186/s12916-025-04432-0 (PMC12590762; doi:10.1186/s12916-025-04432-0)
Supplement: Supplementary file 3 — Additional file 3: STROBE checklist. [file 12916_2025_4432_MOESM3_ESM.docx]

STROBE Statement—Checklist of items that should be included in reports of ***cross-sectional studies***

|  | Item No | Recommendation | Location |
| --- | --- | --- | --- |
| **Title and abstract** | 1 | (*a*) Indicate the study’s design with a commonly used term in the title or the abstract | Abstract, pg 2  Paragraph 2 |
|  |  | (*b*) Provide in the abstract an informative and balanced summary of what was done and what was found | Abstract, pg 2  Paragraph 2-4 |
| Introduction | | |  |
| Background/rationale | 2 | Explain the scientific background and rationale for the investigation being reported | Background, pg 3-4  Paragraphs 1-3 |
| Objectives | 3 | State specific objectives, including any prespecified hypotheses | Background, pg 4  Paragraph 4 |
| Methods | | |  |
| Study design | 4 | Present key elements of study design early in the paper | Methods, pg 4-5  Introductory paragraph |
| Setting | 5 | Describe the setting, locations, and relevant dates, including periods of recruitment, exposure, follow-up, and data collection | Methods  Section 1 – Study populations, pg 5-12 |
| Participants | 6 | (*a*) Give the eligibility criteria, and the sources and methods of selection of participants | Methods  Section 1 – Study populations, pg 5-12 |
| Variables | 7 | Clearly define all outcomes, exposures, predictors, potential confounders, and effect modifiers. Give diagnostic criteria, if applicable | Methods  Section 1 – Study populations (main phenotype & genotype data), pg 5-12  Section 2 – Observational associations, pg 13-15  Section 3 – Fetal genetic scores, pg 15-16 |
| Data sources/ measurement | 8* | For each variable of interest, give sources of data and details of methods of assessment (measurement). Describe comparability of assessment methods if there is more than one group | Methods  Section 1 – Study populations (main phenotype & genotype data), pg 5-12  Section 2 – Observational associations, pg 13-15  Section 3 – Fetal genetic scores pg 15-16 |
| Bias | 9 | Describe any efforts to address potential sources of bias | Methods  Section 3 – Fetal genetic scores  Specifically: adjustment for ancestry principal components and maternal genotype, pg 16, paragraph 4 |
| Study size | 10 | Explain how the study size was arrived at | Methods  Section 1 – Study populations, pg 5-12  Section 2 – Observational associations, pg 13-15  Section 3 – Fetal genetic scores, pg 15-16 |
| Quantitative variables | 11 | Explain how quantitative variables were handled in the analyses. If applicable, describe which groupings were chosen and why | Methods  Section 2 – Observational associations, pg 13-15  Section 3 – Fetal genetic scores, pg 15-16 |
| Statistical methods | 12 | (*a*) Describe all statistical methods, including those used to control for confounding | Methods  Section 1 – Study populations (genotype preparation), pg 5-12  Section 2 – Observational associations, pg 13-15  Section 3 – Fetal genetic scores, pg 15-16 |
|  |  | (*b*) Describe any methods used to examine subgroups and interactions | Methods  Section 2 – Observational associations, pg 13-15  Section 3 – Fetal genetic scores, pg 15-16 |
|  |  | (*c*) Explain how missing data were addressed | Methods  Section 2 – Observational associations (left-censored analysis of the MoBa data – paragraph 3 & complete case analysis – paragraph 4) |
|  |  | (*d*) If applicable, describe analytical methods taking account of sampling strategy | N/A |
|  |  | (*e*) Describe any sensitivity analyses | N/A |
| Results | | |  |
| Participants | 13* | (a) Report numbers of individuals at each stage of study—eg numbers potentially eligible, examined for eligibility, confirmed eligible, included in the study, completing follow-up, and analysed | Methods  Section 1 – Study populations (reports initial eligibility/recruitment numbers and numbers of available genotyped samples), pg 5-12  Results  Table 1, pg 17 (we predominantly report the numbers analysed in our study) |
|  |  | (b) Give reasons for non-participation at each stage | N/A |
|  |  | (c) Consider use of a flow diagram | N/A |
| Descriptive data | 14* | (a) Give characteristics of study participants (eg demographic, clinical, social) and information on exposures and potential confounders | Results, pg 16  Paragraph 1  Table 1, pg 17 |
|  |  | (b) Indicate number of participants with missing data for each variable of interest | N/A |
| Outcome data | 15* | Report numbers of outcome events or summary measures | Results  Table 1, pg 17 |
| Main results | 16 | (*a*) Give unadjusted estimates and, if applicable, confounder-adjusted estimates and their precision (eg, 95% confidence interval). Make clear which confounders were adjusted for and why they were included | Results  Sections 1-3, pg 17-21  Methods  Sections 2-3, pg 13-16 |
|  |  | (*b*) Report category boundaries when continuous variables were categorized | Methods  Section 2 – Observational associations, pg 13-15 |
|  |  | (*c*) If relevant, consider translating estimates of relative risk into absolute risk for a meaningful time period | N/A |
| Other analyses | 17 | Report other analyses done—eg analyses of subgroups and interactions, and sensitivity analyses | N/A |
| Discussion | | |  |
| Key results | 18 | Summarise key results with reference to study objectives | Discussion, pg 21-23  Paragraphs 1-2 |
| Limitations | 19 | Discuss limitations of the study, taking into account sources of potential bias or imprecision. Discuss both direction and magnitude of any potential bias | Discussion, pg 25-27  Paragraphs 7-10 |
| Interpretation | 20 | Give a cautious overall interpretation of results considering objectives, limitations, multiplicity of analyses, results from similar studies, and other relevant evidence | Discussion, pg 23-27  Paragraphs 3-6, 11 (Conclusions) |
| Generalisability | 21 | Discuss the generalisability (external validity) of the study results | Discussion, pg 27  Paragraph 10 |
| Other information | | |  |
| Funding | 22 | Give the source of funding and the role of the funders for the present study and, if applicable, for the original study on which the present article is based | Financial Disclosure/Funding section |

*Give information separately for exposed and unexposed groups.

**Note:** An Explanation and Elaboration article discusses each checklist item and gives methodological background and published examples of transparent reporting. The STROBE checklist is best used in conjunction with this article (freely available on the Web sites of PLoS Medicine at http://www.plosmedicine.org/, Annals of Internal Medicine at http://www.annals.org/, and Epidemiology at http://www.epidem.com/). Information on the STROBE Initiative is available at www.strobe-statement.org.
